# Hepcidin-mediated Iron Regulation in P19 Cells is Detectable by Magnetic Resonance Imaging

**DOI:** 10.1038/s41598-020-59991-4

**Published:** 2020-02-21

**Authors:** Kobra Alizadeh, Qin Sun, Tabitha McGuire, Terry Thompson, Frank S. Prato, Jim Koropatnick, Neil Gelman, Donna E. Goldhawk

**Affiliations:** 1 Imaging, Lawson Health Research Institute, London, Ontario Canada; 2 Medical Biophysics, Western University, London, Ontario Canada; 3 Collaborative Graduate Program in Molecular Imaging, Western University, London, Ontario Canada; 4 Medical Imaging, Western University, London, Ontario Canada; 5 Physics and Astronomy, Western University, London, Ontario Canada; 6 London Regional Cancer Program, London, Ontario Canada; 7 Oncology, Western University, London, Ontario Canada

**Keywords:** Extracellular signalling molecules, Imaging techniques and agents

## Abstract

Magnetic resonance imaging can be used to track cellular activities in the body using iron-based contrast agents. However, multiple intrinsic cellular iron handling mechanisms may also influence the detection of magnetic resonance (MR) contrast: a need to differentiate among those mechanisms exists. In hepcidin-mediated inflammation, for example, downregulation of iron export in monocytes and macrophages involves post-translational degradation of ferroportin. We examined the influence of hepcidin endocrine activity on iron regulation and MR transverse relaxation rates in multi-potent P19 cells, which display high iron import and export activities, similar to alternatively-activated macrophages. Iron import and export were examined in cultured P19 cells in the presence and absence of iron-supplemented medium, respectively. Western blots indicated the levels of transferrin receptor, ferroportin and ubiquitin in the presence and absence of extracellular hepcidin. Total cellular iron was measured by inductively-coupled plasma mass spectrometry and correlated to transverse relaxation rates at 3 Tesla using a gelatin phantom. Under varying conditions of iron supplementation, the level of ferroportin in P19 cells responds to hepcidin regulation, consistent with degradation through a ubiquitin-mediated pathway. This response of P19 cells to hepcidin is similar to that of classically-activated macrophages. The correlation between total cellular iron content and MR transverse relaxation rates was different in hepcidin-treated and untreated P19 cells: slope, Pearson correlation coefficient and relaxation rate were all affected. These findings may provide a tool to non-invasively distinguish changes in endogenous iron contrast arising from hepcidin-ferroportin interactions, with potential utility in monitoring of different macrophage phenotypes involved in pro- and anti-inflammatory signaling. In addition, this work demonstrates that transverse relaxivity is not only influenced by the amount of cellular iron but also by its metabolism.

## Introduction

Inflammation is an immune system response to harmful stimuli, activating immune cells to remove noxious agents and initiate tissue repair^1^. In particular, monocytes are recruited to inflammatory sites and differentiate into pro-inflammatory (M1) and anti-inflammatory (M2) macrophages^2^. M1 (classically-activated) macrophages secrete pro-inflammatory cytokines that facilitate removal of pathogens and/or damaged cells. M2 (alternatively-activated) macrophages are responsible for inflammation resolution and tissue repair^3^.

Iron handling mechanisms differ in these two macrophage phenotypes. M1 macrophages have low ferroportin (FPN, the only recognized iron export protein in vertebrates)^4^ and high ferritin, resulting in low iron export and high iron storage, respectively^5^. Low FPN helps remove pathogens by limiting the availability of iron for bacterial growth. During inflammation, for example, upregulation of the endocrine hormone hepcidin^6^ results in downregulation of FPN and iron retention in M1 macrophages^7,8^. Conversely, M2 macrophages have high FPN and low ferritin, resulting in high iron export and low iron storage, respectively^5^. This facilitates tissue repair by providing iron to adjacent cells, which is a necessary co-factor in inflammation resolution. Being able to distinguish between M1 and M2 macrophages *in vivo* may lead to a better understanding of the different phases of inflammation and improve diagnosis and treatment outcomes^9–11^.

MRI is a non-invasive imaging method that can be used to track cellular activities involved in different diseases. Toward achieving molecular imaging capability, various iron-based exogenous and endogenous contrast agents have been developed to enhance image contrast and improve molecular imaging^12,13^. In addition, cellular iron metabolism might also be expected to influence the accumulation of contrast agents and their detection by MRI^14^. In the case of iron-exporting cells (particularly pro- and anti-inflammatory macrophages), little is known about how their distinct iron regulation may be distinguished by MRI. To investigate this, we used the multi-potent P19 stem cell model with high iron import and export activities^15^, the latter of which corresponds with high FPN^14^. In this regard, P19 cells resemble macrophages^5^ and are a convenient model of iron regulation related to inflammation signaling. We examined the effect of varying extracellular iron supplementation and hepcidin on MR contrast in undifferentiated P19 cells and confirmed that changes in total cellular iron content were accompanied by changes in the level of FPN and transverse relaxation rates. In addition, we showed that hepcidin regulation of FPN is active in the P19 cell line and influences the correlation between total cellular iron and transverse relaxivity.

## Materials and Methods

### Cell model

Mouse multi-potent teratocarcinoma cells (P19, ATCC CRL-1825) were cultured in α-minimum essential medium (α-MEM) supplemented with 10% fetal bovine serum, 4 U/mL penicillin and 4 μg/mL streptomycin. Cells were incubated in a humid chamber at 37 °C with a 5% CO_2_/air mixture and passaged 1:10 when they reached 70% confluency. Cells were harvested by trituration alone for protein expression analysis or after 30 sec incubation with 0.05% Trypsin/EDTA for trace element analysis and MR relaxation rate measurements. All cell culture reagents were purchased from Life Technologies, Burlington, Canada.

### Iron supplementation

**Figure 1:**
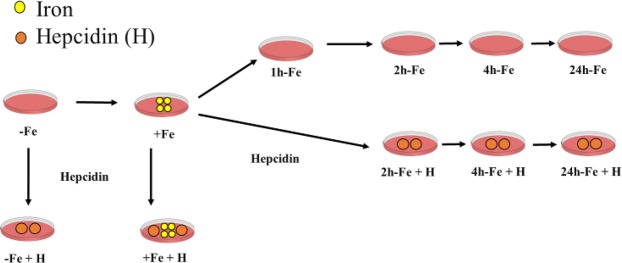
Flow chart of P19 cell sample preparation. Cells were cultured in non-supplemented (−Fe) or iron-supplemented (+Fe) medium containing 25 µM ferric nitrate for at least 5–7 days prior to withdrawal of iron supplementation and further culture in non-supplemented medium for an additional 1 (1h-Fe), 2 (2h-Fe), 4 (4h-Fe) or 24 (24h-Fe) hours (first row). To examine the response to hepcidin, 200 ng/mL hepcidin/medium was added to the culture immediately after removal of iron supplementation (second row). In addition, −Fe and +Fe samples were incubated with hepcidin for the last 24 hours of culture in non-supplemented (−Fe + H) or iron-supplemented (+Fe + H) medium, respectively. At each time point, live cells were harvested and either prepared for MRI or lysed and analyzed by western blot and inductively-coupled plasma mass spectrometry (ICP-MS).

A flow chart depicting sample preparation is shown in Fig. 1. Published methods were adapted to study the P19 cell response to extracellular iron^15^. Accordingly, cells were cultured in the absence (−Fe) or presence (+Fe) of an iron supplement: 25 µM ferric nitrate (Sigma-Aldrich, Oakville, Canada)/medium for at least 5–7 days. After iron supplementation, extracellular iron was removed and replaced with non-supplemented medium for an additional 1 (1h-Fe), 2 (2h-Fe), 4 (4h-Fe) and 24 (24h-Fe) hours, to examine iron export activity in P19 cells over time (Fig. 1, first row). Changes in total cellular iron content, FPN expression and MR signal were explored over the treatment timeframe, as described below.

### Hepcidin treatment

To investigate the effect of hepcidin, cells were cultured in medium containing 200 ng/mL hepcidin^16^ (Sigma-Aldrich, Oakville, Canada), added after the removal of extracellular iron supplement (Fig. 1, second row). Cell samples +/−Fe supplement were also incubated with hepcidin for the last 24 hours of culture. This was performed to separate potential changes in the cellular response to inflammation (*i*.*e*. hepcidin) from those changes arising from the combination of hepcidin and increased extracellular iron (*i*.*e*. hemorrhage).

### Protein sample preparation

Cells were cultured under different conditions of iron supplementation and hepcidin treatment as described above (Fig. 1); then washed twice using 10 mL phosphate buffered saline (PBS, 137 mM NaCl/2.7 mM KCl/10 mM Na_2_HPO_4_); and collected in 1 mL radioimmunoprecipitation assay buffer (RIPA; 10 mM Tris-HCl pH 7.5/140 mM NaCl/1% NP-40/1% sodium deoxycholate/0.1% sodium dodecyl sulfate [SDS]) containing 150 μL Complete Mini protease inhibitor cocktail (Roche Diagnostic Systems, Laval, Canada). Thorough cell lysis was accomplished by sonication, using three 10 sec bursts of a Sonic Dismembrator (model 500, Fischer Scientific) at an amplitude of 29%. Total amount of protein was quantified using the BCA assay^17^.

### Western blot

All reagents were purchased from Thermo Fisher Scientific, Mississauga, Canada, unless otherwise indicated. Western blots were prepared by adapting published procedures^18^. Briefly, samples containing 20 µg total cellular protein were reduced with 1 mM dithiothreitol (DTT) and heated at 80 °C for 5 min prior to discontinuous SDS polyacrylamide gel electrophoresis (SDS-PAGE) using a 10% running gel. Protein was transferred onto a nitrocellulose blot using the Original iBlot Gel Transfer Device (Life Technologies, Burlington, Canada). Nonspecific protein binding was blocked in 5% bovine serum albumin (BSA)/Tris-buffered saline pH 7.4 (TBS)/0.02% sodium azide (TBSA) for a minimum of 2 h at room temperature. For FPN detection, blots were incubated 18 h in 1:1000 rabbit α-FPN1/5% BSA/TBSA at 4 °C; then washed using TBS/0.1% Tween 20 (TBST, Sigma-Aldrich, Oakville, Canada) for 30 min with 4 changes of buffer; and incubated 1 h in 1:20,000 horseradish peroxidase (HRP)-conjugated goat α-rabbit IgG (Sigma-Aldrich, Oakville, Canada)/5% BSA/TBS at room temperature. Finally, blots were washed with 0.1% TBST for 30 min with 2 changes of buffer and imaged using the Chemigenius Gel Doc (Syngene). A chemiluminescent signal was detected using SuperSignal West Pico Chemiluminescent Substrate according to the manufacturer’s instructions.

For ubiquitin detection, primary mouse α-Ub (BioLegend, San Diego, USA) was used at 1:1000. For transferrin receptor detection, primary rabbit α-TfRc (NovusBio, Oakville, Canada) was used at 1:1000. The reported molecular weight (MW) for FPN is approximately 63 K^19,20^, for ubiquitin is approximately 150 K^21^, and for transferrin receptor is approximately 89 K^22^. Glyceraldehyde 3-phosphate dehydrogenase (GAPDH, approximately 37 K)^23^ served as a loading control. For GAPDH detection, blots were stripped and reprobed as detailed above, with the following changes: primary incubation in 1:2000 rabbit α-GAPDH and secondary incubation in 1:20,000 HRP-conjugated goat α-rabbit IgG, all at room temperature.

To assess changes in expression, the signal intensity of each band was normalized to the corresponding GAPDH band for that sample, using Image Lab software version 5.2. Results were then normalized to the +Fe (no hepcidin) condition. Representative blots were selected from the analysis of 3 sets of samples consisting of biological replicates.

### Trace element analysis

At harvest, P19 cells were lysed and protein quantified as described above. The total amount of elemental iron was measured by ICP-MS (Biotron Analytical Services, Western University) and normalized to amount of protein. Data are the mean +/− SEM for n = 3–20.

### Cell harvest and MR phantom preparation

**Figure 2:**
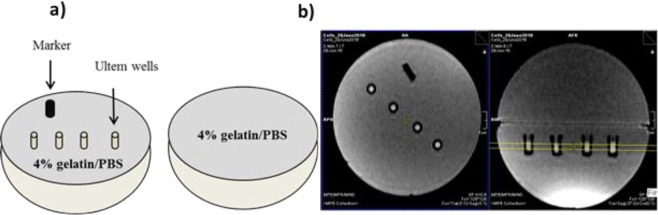
MRI cell phantom and slice localization. (**a**) Two hemispheres of a 9 cm plastic mold were filled with 4% gelatin/PBS. Cells were placed in Ultem wells in one hemisphere, overlaid with 4% gelatin/PBS and covered by the other hemisphere. Sample orientation was indicated by a plastic peg. (**b**) Using a knee radiofrequency (RF) coil, images were acquired at 3 Tesla (3T). In the cross-sectional view (left panel), the arrangement of sample wells is shown. A 3-mm thick slice perpendicular to the wells was defined for image acquisition in the sagittal view (right panel, yellow box).

P19 cells were cultured in 150 mm culture dishes to obtain 40–50 million cells. At harvest, cells were centrifuged at 400 × g for 5 min at 15 °C, repeating as needed to obtain the desired compact cell pellet in custom made Ultem wells (inner diameter, 4 mm; height, 10 mm; Lawson Imaging Prototype Lab). Afterwards, wells were placed in a 9 cm, spherical 4% gelatin (porcine type 1, Sigma-Aldrich, Oakville, Canada)/PBS phantom^24^ and overlaid with sterile filtered 4% gelatin/PBS (Fig. 2a).

### Data acquisition and relaxation rate calculation

Using a knee radio frequency (RF) coil, the gelatin phantom was scanned on a 3 Tesla (3T) mMR Biograph (Siemens AG, Erlangen, Germany), using previously described sequences^24^ to obtain transverse relaxation rates. A multi-echo gradient echo sequence was applied to obtain the total transverse relaxation time (*T*_2_*) and its associated rate (*R*_2_* = 1/*T*_2_*). A single-echo spin echo sequence was applied to obtain the irreversible component of transverse relaxation time (*T*_*2*_) and its associated rate (*R*_*2*_ = 1/*T*_*2*_). The reversible component, ${R}_{2}^{{\prime} }$, was calculated by subtraction (*R*_2_* − *R*_*2*_). Data are the mean +/− SEM for n = 3–9.

The following imaging parameters were employed. For the single-echo spin echo sequence: echo time (TE) = 13, 30, 40, 60, 80, 100, 150, 200, 300 ms; repetition time (TR) = 2010 ms; flip angle = 90°; total scanning time = approximately 61 min. For the multi-echo gradient echo sequence: TE = 6.12, 14.64, 23.16, 31.68, 40.2, 50, 60, 70, 79.9 ms; TR = 200 ms; flip angle = 60°; total scanning time = approximately 25 min. In both sequences, the field of view was 120 × 120 mm^2^, the voxel size was 1.5 × 0.6 × 0.6 mm^3^ and the matrix size was 192 × 192. The slice thickness was 3 mm, perpendicular to the wells (Fig. 2b).

### Region of interest (ROI) and relaxation rate measurements

Analysis software was previously developed in Matlab 7.9.0 (R2010b)^15,24^ and used to determine ROI and to measure *R*_2_* and *R*_*2*_. ROI was outlined to include as many voxels as possible within the sample wells while avoiding the wall of the wells. Approximately 21 voxels were included in the circular ROI. Relaxation rates were determined using the average signal intensity for each time point and least-squares curve fitting.

### Statistics

Mean and standard error of the mean (SEM) were calculated. Two-way analysis of variance (ANOVA) was performed to examine any significant differences among treatment groups (p < 0.05). Pearson’s correlation was applied to examine potential correlation between cellular iron and relaxation rates and the regression model identified the best linear equation between each relaxation rate as dependent variable and cellular iron content as independent variable. To compare the slopes of linear correlations, student’s t-test was performed. The strength of the correlations was compared using Fisher Z transformation. All statistical analyses were performed using IBM SPSS Statistics, version 25. All graphs were created using the GraphPad Prism package, version 7.03.

## Results

### P19 response to extracellular iron supplementation

#### Analysis of intracellular iron content

Iron import and export activities in P19 cells were investigated using various conditions of iron supplementation. To examine iron import activity, cells were cultured in the absence (−Fe) or presence (+Fe) of extracellular iron supplementation (25 µM ferric nitrate/medium) for at least 5–7 days. To examine iron export activity, changes in total cellular iron content arising within hours of the withdrawal of iron supplement were examined. Accordingly, iron-supplemented P19 cells were harvested either immediately or after additional culture in non-supplemented medium for 1, 2, 4 or 24 hours (Fig. 1, first row).

**Figure 3:**
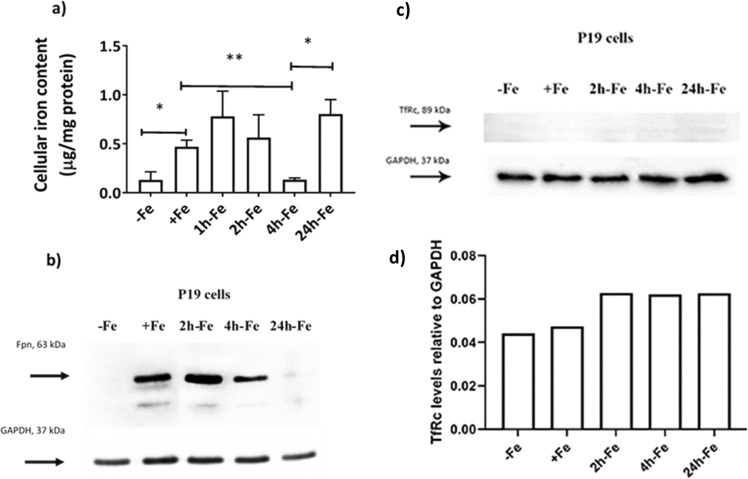
Iron handling in P19 cells under various conditions of extracellular iron supplementation. Cells were cultured either in non-supplemented medium (−Fe) or iron-supplemented medium (+Fe) for at least 5–7 days before iron supplementation withdrawal and an additional 1 (1h-Fe), 2 (2h-Fe), 4 (4h-Fe) or 24 (24h-Fe) hours of culture in non-supplemented medium. (**a**) Total cellular iron content was measured by ICP-MS and normalized to total amount of protein. Data are the mean ± SEM (*p < 0.05, **p < 0.01, ***p < 0.001): −Fe, n = 4; +Fe, n = 9; 1h-Fe, n = 3; 2h-Fe, n = 3; 4h-Fe, n = 3; 24h-Fe, n = 20. (**b**) Protein lysates from P19 cells were examined by western blot, probed with α-FPN 1 (top panel) and α-GAPDH (bottom panel). Approximate MW is indicated in the left margin. Full-length blots are presented in Supplementary Fig. S1. (**c**) Similar immunoblots were also probed with α-TfRc (top panel) and α-GAPDH (bottom panel). (**d**) The signal intensity of each TfRc band was normalized to the corresponding GAPDH band, revealing little or no change in the level of TfRc. Full-length blots are presented in Supplementary Fig. S2.

Figure 3a shows the mean values of total cellular iron content, as determined by ICP-MS and normalized to amount of protein. Based on trace element analysis, cellular iron content increased (p < 0.05) after iron supplementation (+Fe) compared to samples cultured in non-supplemented medium (−Fe), confirming high iron import activity in P19 cells. However, upon removal of iron supplementation and continued culture in non-supplemented medium, cellular iron content decreased, reaching baseline values after 4 hours (+Fe vs 4h-Fe, p < 0.01), suggesting high iron export activity. Interestingly, cellular iron level increased again, 24 hours after removal of iron supplement (4h-Fe vs 24h-Fe, p < 0.05).

### Ferroportin levels

Possible changes in FPN protein levels in response to extracellular iron supplementation were examined by western blot (Fig. 3b). Prior to harvest, cells were cultured in the presence or absence of extracellular iron supplementation (Fig. 1, first row). The relatively constant level of GAPDH gave a uniform band in each lane, as determined by densitometry (data not shown, refer to changes +/− hepcidin and Supplementary Fig. S1).

A comparison of FPN and total cellular iron content indicates that P19 cells upregulate iron export in response to extracellular iron. When this iron supplement is withdrawn, FPN remains elevated until intracellular iron stores return to baseline values, after approximately 4 hours. Interestingly, when FPN level returns to baseline at 24h-Fe, cellular iron content rises sharply despite the absence of an extracellular iron supplement. The +Fe samples indicate active iron import, presumably through TfRc. Figure 3c,d indicate the status of TfRc and show little or no difference between samples (refer to full-length blots in Supplementary Fig. S2), consistent with a dominant role for FPN regulation in maintaining iron homeostasis. In summary, FPN in P19 cells is influenced by the presence of both extracellular and intracellular iron.

### MRI relaxation rates

**Figure 4:**
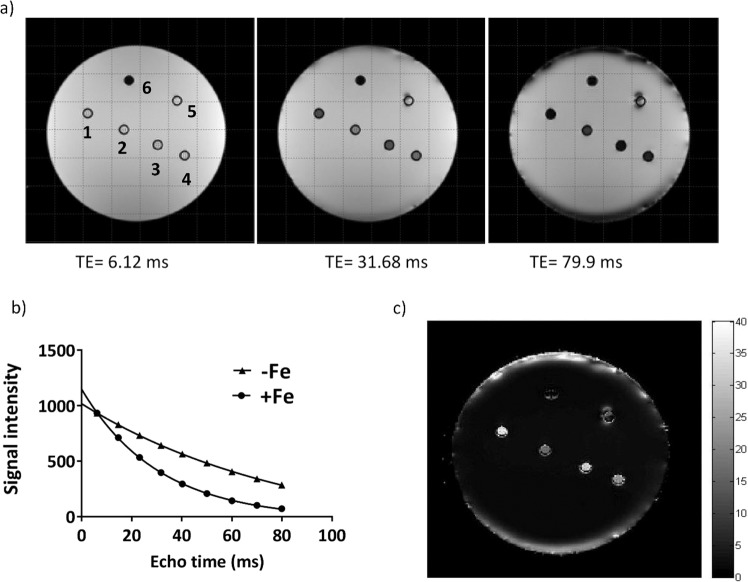
Transverse relaxation rate measurement and mapping in the spherical phantom. (**a**) Signal intensity decreases over time in a *T*_2_*-weighted image of a representative phantom set up. Sample wells are labelled counter clockwise from 1 to 5, indicating +Fe, −Fe, 24h-Fe, 24h-Fe and +Fe + H, respectively. Number 6 shows the plastic peg for reference. (**b**) Signal decay curves are shown for +Fe and −Fe conditions. Each point shows the mean signal intensity measured within the defined ROI. The best fit for an exponential decay is shown by each curve. Iron supplementation resulted in an increase in *R*_2_* (1/*T*_2_*). (**c**) The *R*_2_* map illustrates a representative phantom. The map was obtained using voxel by voxel curve fitting with an exponential decay function. Higher *R*_2_* is observed for the +Fe condition (1) compared to −Fe (2).

**Figure 5:**
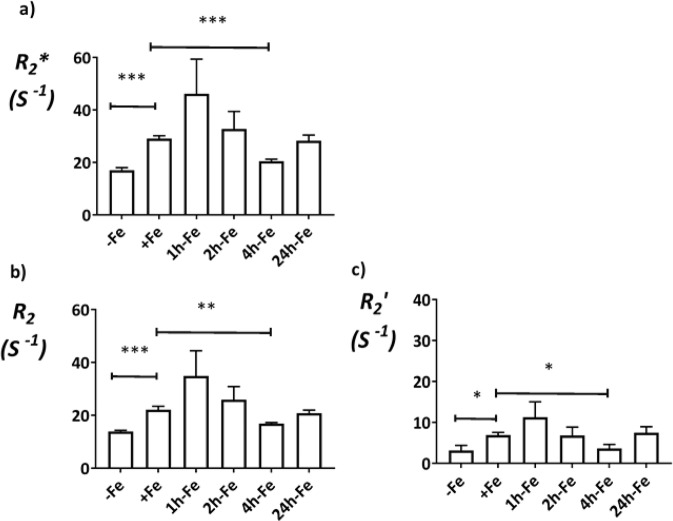
Transverse relaxation rates of P19 cells under various conditions of extracellular iron supplementation. Cells were cultured either in non-supplemented medium (−Fe) or iron-supplemented medium (+Fe) for at least 5–7 days before iron supplementation withdrawal and an additional 1 (1h-Fe), 2 (2h-Fe), 4 (4h-Fe) or 24 (24h-Fe) hours of culture in non-supplemented medium. (a) *R*_2_* and (b) *R*_*2*_ were determined at 3T and (c) ${R}_{2}^{\text{prime}}$ was calculated for each sample: ${R}_{2}^{\text{prime}}$ = *R*_2_* − *R*_*2*_. An increase in each transverse relaxation rate was observed after iron supplementation, consistent with active iron import in P19 cells. Within 4 hours of the withdrawal of extracellular iron supplement, the signal returned to baseline, consistent with an increase in iron export protein. This finding substantiates dynamic iron regulation in P19 cells. Data are the mean ± SEM (*p < 0.05; **p < 0.01; ***p < 0.001; −Fe, n = 4; +Fe, n = 9; 1h-Fe, n = 3; 2h-Fe, n = 3; 4h-Fe, n = 3; 24h-Fe, n = 9).

To examine possible changes in MR relaxation rates under various conditions of iron supplementation (Fig. 1, first row), cells were harvested and scanned at 3T using a spherical gelatin phantom. Figure 4a shows the signal intensity map of a representative phantom set-up for three different TE values in the *T*_2_*-weighted image. The corresponding signal decay curves for +Fe and −Fe samples in the phantom (denoted by numbers 1 and 2, respectively) are shown in Fig. 4b. The *R*_2_* map is shown in Fig. 4c. Mean values of transverse relaxation rates are shown in Fig. 5. Relaxation rate measurements showed the same pattern as observed with total cellular iron content (Fig. 3a) over the treatment timeframe. As shown in Fig. 5a, *R*_2_* increased after iron supplementation compared to untreated cultures (−Fe vs +Fe, p < 0.001), consistent with the avid iron import activity in P19 cells reported in a previous study^15^. Upon removal of extracellular iron supplementation, *R*_2_* decreased to baseline levels within 4 hours (+Fe vs 4h-Fe, p < 0.001), consistent with reported iron export activity in P19 cells^15^ and clarifying the time course of iron export. While similar to the pattern observed in cellular iron content (Fig. 3a), an increase in *R*_2_* at 24h-Fe fell short of statistical significance (4h-Fe vs 24h-Fe, p = 0.070, n = 9).

As previously described, the total transverse relaxation rate, *R*_2_* consists of two components: *R*_*2*_ and ${R}_{2}^{\text{prime}}$. The same comparisons were investigated for each component (Fig. 5b,c). Although different in magnitude, each component of the transverse relaxation rate was increased by an extracellular iron supplement (for *R*_*2*_, −Fe vs +Fe, p < 0.001; for ${R}_{2}^{\text{prime}}$, −Fe vs +Fe, p < 0.05) and was decreased within 4 hours of its withdrawal (for *R*_*2*_, +Fe vs 4h-Fe, p < 0.01 and for *R*_*2*_′, +Fe vs 4h-Fe, p < 0.05), falling short of a significant increase at 24h-Fe (for *R*_*2*_, 4h-Fe vs 24h-Fe, p = 0.080 and for *R*_*2*_′, 4h-Fe vs 24h-Fe, p = 0.058).

### P19 response to hepcidin treatment

#### Analysis of intracellular iron content

**Figure 6:**
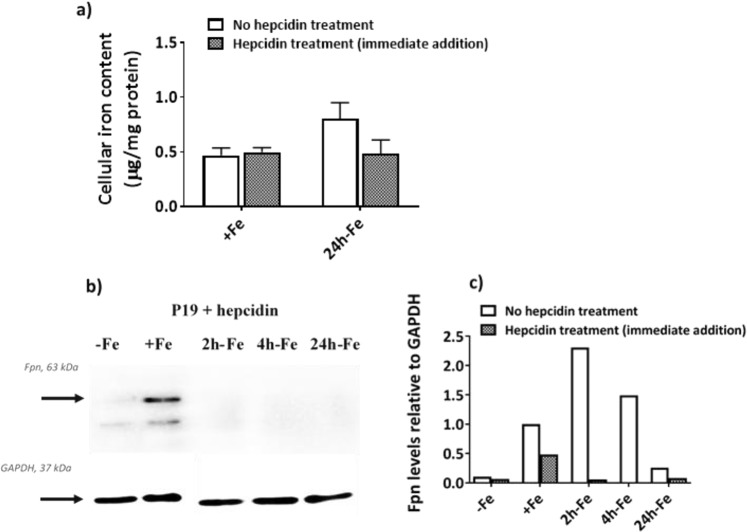
Cellular iron handling in P19 cells in response to hepcidin treatment. Cells were cultured either in non-supplemented medium (−Fe) or iron-supplemented medium (+Fe) for at least 5–7 days before iron supplementation withdrawal and an additional 2 (2h-Fe), 4 (4h-Fe) or 24 (24h-Fe) hours of culture in non-supplemented medium, with or without hepcidin. In the case of hepcidin treatment +/−Fe, cells were incubated with hepcidin for the last 24 hours of culture. (**a**) Total cellular iron content for +Fe and 24h-Fe samples was measured by ICP-MS and normalized to total amount of protein for samples treated with (gray bars) or without (white bars) hepcidin. No difference was observed between treatment groups: +Fe (no hepcidin), n = 9; +Fe (hepcidin treatment), n = 6; 24h-Fe (no hepcidin), n = 20; 24h-Fe (hepcidin treatment), n = 3. (**b**) Total cellular protein from hepcidin treated cells was examined by western blot, probing with α-FPN 1 (top panel) and α-GAPDH (bottom panel). Approximate MW is shown in the left margin. (**c**) The signal intensity of each FPN band, obtained in the presence and absence (Fig. 3b) of exogenous hepcidin, was normalized to the corresponding GAPDH band. The signal intensity of FPN at 4h-Fe with hepcidin was below the detection limit. All ratios were subsequently normalized to the +Fe (no hepcidin) condition. Overall, FPN was downregulated in response to hepcidin. Full-length blots are presented in Supplementary Fig. S1.

Figure 6a summarizes the effect of hepcidin on total cellular iron content under two conditions in which iron uptake is evident but the level of iron export protein (hepcidin’s target) is different (Fig. 3b). Cells were cultured in the presence of 25 µM ferric nitrate/medium for at least 5–7 days (+Fe) and then incubated with or without 200 ng/mL hepcidin (H, Fig. 1) for the last 24 hours of culture in either iron supplemented medium (+Fe vs +Fe + H) or after removal of iron supplement (24h-Fe vs 24h-Fe + H). The aim was to explore if any further increase in cellular iron content was achievable beyond the +Fe or 24h-Fe condition (refer to Fig. 3a,b) by blocking iron export activity in these cells. As shown in Fig. 6a, no difference was observed in total cellular iron content between hepcidin-treated and untreated cells. A similar result was obtained for samples at 2h-Fe and 4h-Fe (Supplementary Fig. S5a).

### Ferroportin levels

To examine the potential for hepcidin to regulate iron export in P19 cells, cultures were supplemented with iron for at least 5–7 days (+Fe) thereby stimulating FPN expression (Fig. 3b). Cells were harvested after withdrawal of iron supplement and culture in non-supplemented medium for an additional 2 (2h-Fe), 4 (4h-Fe) and 24 (24h-Fe) hours in the presence of 200 ng/mL hepcidin (Fig. 1, second row). In addition, cells incubated in non-supplemented medium (−Fe) and in continuously iron-supplemented medium (+Fe) were treated with hepcidin for the last 24 hours of their culture (Fig. 1). Western blots were used to detect potential changes in FPN in response to hepcidin treatment and compared to non-hepcidin treated cells. As shown in Fig. 6b,c, FPN immunostaining decreased in the presence of hepcidin (2h-Fe and 4h-Fe). Also, densitometric analysis comparing Figs. 3b and 6b showed that, after hepcidin treatment, FPN immunostaining decreased in the continuously iron-supplemented sample (+Fe + H) by approximately 50% (refer to full-length blots in Supplementary Fig. S1). This finding is consistent with distinct pathways for iron-stimulated expression of FPN and hepcidin-meditated degradation of FPN^25^. As expected, the low level of FPN expression in −Fe and 24h-Fe samples did not change in the presence of hepcidin.

To better understand the iron regulatory capabilities of P19 cells, we examined the levels of ubiquitin in the presence and absence of hepcidin treatment. Western blots revealed upregulation of ubiquitin in response to exogenous hepcidin (Supplementary Figs. S2 and S3), consistent with ubiquitin-mediated degradation of FPN. When the 150 K ubiquitin band was compared to GAPDH using densitometry, the ratios of ubiquitin/GAPDH were increased in hepcidin-treated samples compared to no hepcidin treatment (Table 1).

**Table 1:** Ubiquitin levels in P19 cells respond to hepcidin.

Iron Supplementation	Hepcidin Treatment	^a^Ratio of Ubiquitin/GAPDH		^b^Mean Ratio Ubiquitin/GAPDH (n = 8)	SD	*p* value
		Sample 1	Sample 2			
+Fe	−	1	1	1.23	0.53	<0.01
2h-Fe	−	2.64	0.95			
4h-Fe	−	2.05	1.01			
24h-Fe	−	0.45	0.75			
+Fe	+	6	2.36	3.27	0.60	
2h-Fe	+	4.23	1.56			
4h-Fe	+	4.45	1.45			
24h-Fe	+	4.32	1.82			

^a^Ratios were normalized to the iron-supplemented sample (+Fe) not treated with hepcidin (−).

^b^Mean ratio of Ubiquitin/GAPDH in hepcidin-treated (+) and untreated samples were compared using an unpaired Student’s t-test.

### MRI relaxation rates

Similar to the influence of hepcidin on cellular iron content, +Fe and 24h-Fe conditions summarize the effect of hepcidin on MR transverse relaxation rates. Cells were incubated in the presence or absence of hepcidin (Fig. 1, second row) and then scanned at 3T to measure transverse relaxation rates (Supplementary Fig. S4). No difference in any of the transverse relaxation rates was observed between hepcidin-treated and control P19 cells. The same was true at 2h-Fe and 4h-Fe (Supplementary Fig. S5b–d). Thus, the magnitude of the MR signal was not influenced by hepcidin-mediated regulation of FPN.

### Correlation between MR signal and cellular iron content

**Figure 7:**
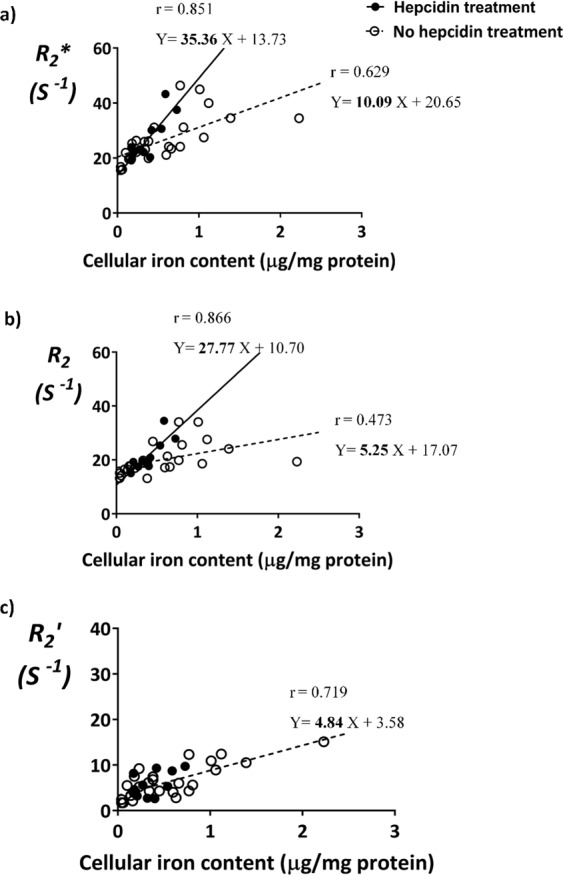
Comparison of MR relaxation rates and total cellular iron content in P19 cells. Cells were cultured in the absence (empty circles, n = 25) or presence (filled circles, n = 11) of hepcidin, with or without extracellular iron supplementation or its withdrawal, as described in Fig. 1. Total cellular iron content was determined by ICP-MS and normalized to total cellular protein. Transverse relaxation rates were obtained at 3T. Pearson’s correlation and regression analysis were applied to investigate the relationship between relaxation rates and cellular iron content. Hepcidin treatment increases slopes of the line relating *R*_2_* vs cellular iron and *R*_*2*_ vs cellular iron. However, no linear relationship was found between ${R}_{2}^{\text{prime}}$ vs cellular iron after hepcidin treatment.

While FPN level was altered, non-significant differences in total cellular iron content and transverse relaxation rates, before and after hepcidin treatment, led us to examine whether the correlation between MR signal and cellular iron content had been influenced by hepcidin. Having presented the variation (mean +/− SEM) in both transverse relaxation rates (Figs. 5 and S4) and elemental iron content (Figs. 3a and 6a), only samples with matching data were used for the correlation. Accordingly, samples were separated into their respective treatment groups: hepcidin vs no hepcidin. Pearson’s correlation test was applied to investigate the relationship between cellular iron content as the independent variable and transverse relaxation rate as the dependent variable. In the absence of hepcidin (Fig. 7, open circles; Table 2), there is a moderate correlation between *R*_2_* and cellular iron content (r = 0.629, p < 0.001), a weaker correlation between *R*_*2*_ and cellular iron content (r = 0.473, p < 0.01) and a strong correlation between ${R}_{2}^{\text{prime}}$ and cellular iron content (r = 0.719, p < 0.001). On the other hand, in the presence of hepcidin (Fig. 7, filled circles; Table 2), a strong correlation was observed between *R*_2_* and cellular iron content (r = 0.851, p < 0.001) and between *R*_*2*_ and cellular iron content (r = 0.866, p < 0.001). However, the correlation between ${R}_{2}^{\text{prime}}$ and cellular iron content was only moderate (r = 0.532, p < 0.05).

**Table 2:** Correlation between MR relaxation rates and total cellular iron content in P19 cells.

Transverse relaxation rates	^a^Whole data set (n = 36)	No hepcidin treatment (n = 25)			Hepcidin treatment (n = 11)			Difference between slopes
	^b^r	r	^b^β	r^2^	r	β	r^2^	
*R*_2_*	0.607 p < 0.001	0.629 p < 0.001	**10**.**09**	0.396	0.851 p < 0.001	**35**.**36**	0.724	**p** < **0**.**05**
*R*_*2*_	0.468 p < 0.01	0.473 p < 0.01	**5**.**25**	0.224	0.866 p < 0.001	**27**.**77**	0.750	**p** < **0**.**01**
${R}_{2}^{\text{prime}}$	0.679 p < 0.001	0.719 p < 0.001	4.84	0.517	0.532 p < 0.05	^c^n.s.	n.s.	^c^n/a

^a^Statistical analysis of the whole data set combines samples treated with (n = 11) and without (n = 25) hepcidin. n, sample size.

^b^r, Pearson correlation coefficient; p, level of significance; β, linear regression slope.

^c^n.s., not significant; n/a, not applicable.

The line-of-best-fit for hepcidin-treated (solid lines) and control (dashed lines) cells was determined using a linear regression model. The difference between slopes was determined using an independent samples t-test. Comparing slopes of the lines for *R*_2_* vs cellular iron (Fig. 7a) revealed an increase from 10.09 for control cells to 35.56 for hepcidin-treated cells (p < 0.05). The same analysis for *R*_*2*_ vs cellular iron (Fig. 7b) showed an increase in slope from 5.25 for control cells to 27.77 for hepcidin-treated cells (p < 0.01). However, for ${R}_{2}^{\text{prime}}$ vs cellular iron (Fig. 7c), no line-of-best-fit was drawn for the hepcidin treatment group due to lack of a significant linear correlation, while the linear correlation for control cells was significant (Table 2, p < 0.001). Taken together, these data suggest that transverse relaxivity may be enhanced by the form of iron present in cells responding to hepcidin-mediated signaling.

## Discussion

Hepcidin is a hepatic hormone which is upregulated in response to inflammation and changes in the level of circulating iron^7,8^. Alterations in MR transverse relaxation rates, total cellular iron content and levels of the iron export protein FPN were investigated in response to hepcidin, using multi-potent mouse P19 embryonic teratocarcinoma cells, cultured under various conditions of extracellular iron supplementation. The results revealed that hepcidin-dependent alterations in iron homeostasis are detectable by MRI. In addition, intracellular iron content was regulated in a manner consistent with the response to changes in extracellular iron and FPN levels.

### Intracellular iron analysis

Iron uptake by mammalian cells occurs mainly through transferrin-transferrin receptor interactions in which transferrin-bound iron is internalized by receptor-mediated endocytosis^26,27^. While iron is a vital element for cellular homeostasis, excess iron causes oxidative damage by creating reactive oxygen species. As a result, in most cells the level of iron uptake is balanced by regulation of transferrin receptor expression^26,28^. When internalized iron is not immediately used, it is mainly sequestered in the storage protein ferritin, as a biomineral with paramagnetic properties^29^. In select cells, iron is exported by FPN, the sole iron export protein identified in vertebrates^25,30^. In the P19 cell model, total cellular iron content increased in response to an extracellular iron supplement, indicating effective iron internalization at relatively low iron concentration. Similar to alternatively-activated macrophages derived from mouse bone marrow^5^, P19 cells display an iron recycling phenotype with substantial iron import and export activity. Even though withdrawal of iron supplement from P19 cell culture was accompanied by a decrease in total cellular iron content within 4 hours, a second rise in cellular iron occurred within 24 hours. While there is little or no change in TfRc level to account for this finding, our results point to the sensitive regulation of iron export protein in response to changes in extracellular iron.

Cellular iron distribution following extracellular iron supplementation has been studied in conjunction with MRI using non-radioactive iron isotopes to distinguish between pre-existing iron and extracellular supplement^31^. Masthoff and colleagues used both ICP-MS of homogenized tissue and laser ablation ICP-MS (LA-ICP-MS) of tissue slices to quantify endogenous iron (^56^Fe) and differentiate it from ^57^Fe incorporated through uptake of an iron oxide nanoparticle. Similar approaches may shed light on the metabolic fate of extracellular iron in the P19 cell model and potentially clarify the effect of iron-related stimuli on hepcidin activity and vice versa. Since iron export activity is a relatively unique feature of monocytes and macrophages, there may be diagnostic value in monitoring hepcidin-ferroportin interactions during inflammation. For example, both hemorrhage and lingering inflammation are present in the heart post-myocardial infarction and implicated in heart failure^32^. In this context, when tissue macrophages, both resident and recruited, are present in sufficient quantity, their distinct iron regulation may permit high resolution techniques like LA-ICP-MS to identify regions that are targeted by immune cell activity. This could help explain why the remote myocardium, distal from the point of infarct, is affected by unresolved inflammation.

Hepcidin triggers internalization and downregulation of FPN in the reticuloendothelial system^16,33–35^. As a consequence, cellular iron accumulation coincides with an increase in ferritin level. However, we compared total cellular iron content in P19 cells treated with and without hepcidin and found no significant change in iron. This may be attributed to the elemental analysis of cellular iron which includes all forms of iron and does not distinguish fluctuations in ferritin or the labile iron pool (LIP). Internalized iron first enters the LIP before being stored in ferritin as Fe (III) or exported by FPN. Although the LIP represents a small fraction of the total cellular iron content under quiescent conditions, this may be dramatically altered in response to biochemical stimuli^36–38^. In P19 cells, hepcidin-associated decreases in iron export may influence LIP composition: an increase in ferritin level might be balanced by a decrease in LIP, which ultimately results in a constant level of total cellular iron content. As discussed below, further analysis of intracellular iron composition may bear on the MR signals detected in the P19 cell model of iron export.

### Protein analysis

FPN is expressed by only a few cell types, including macrophages, enterocytes, hepatocytes and breast epithelia^10,39,40^. Macrophages have a principal role in phagocytosis of damaged or senescent red blood cells, exporting microgram quantities of iron back into plasma for the synthesis of new erythrocytes. This iron recycling proceeds through a FPN-mediated pathway. FPN upregulation in macrophages after iron supplementation and erythrophagocytosis has been shown in several studies^5,34,41,42^. A similar pattern was observed in P19 cells after administering an extracellular iron supplement. On the other hand, FPN downregulation has been reported when macrophages were exposed to desferrioxamine mesylate (DFO)^5,42^, which binds iron particles and deprives the cell of iron similar to withdrawal of extracellular iron from culture. Under the latter conditions, we observed a decrease in FPN levels, again suggesting that characteristics of iron homeostasis in P19 cells may be relevant to macrophage function.

The downregulation of FPN 24 hours after removal of iron supplementation suggests that post-translational regulation of FPN may be active in P19 cells. There are two known mechanisms for post-translational regulation of FPN. Its downregulation in the absence of multicopper oxidases has been reported^43^. The second known mechanism is hepcidin-dependent downregulation of FPN, which raises the possibility of hepcidin production by P19 cells to self-regulate FPN expression as reported for human monocytes^8^.

During inflammation, FPN is post-translationally downregulated in macrophages by the hormone hepcidin^25,44^, which itself is upregulated by inflammation and serum iron^6,45,46^. Hepcidin-dependent internalization and downregulation of FPN have been shown in HEK293 cells expressing mouse FPN^16,35,47^, mouse primary bone marrow-derived macrophages^35,41^ and the mouse macrophage cell line J774^34^. Our results confirmed downregulation of FPN in P19 cells in response to hepcidin, suggesting that this cell line may be a suitable model for further investigation of hepcidin-dependent FPN regulation.

Anti-inflammatory (M2) macrophages exhibit high levels of FPN, resulting in an iron recycling phenotype. This pattern of iron export is also displayed by tumor-associated macrophages (TAM)^48^ and provides a ready supply of iron for uncontrolled tumour growth. On the other hand, pro-inflammatory (M1) macrophages express minimal FPN and represent an iron storage phenotype^5,40^. In this context, the parental P19 cell line shows similar iron handling activities and FPN levels as M2 macrophages and TAM. Consistent with this, P19 are a rapidly growing cell type, doubling in less than 24 hours. This characteristic may be facilitated by their iron recycling ability. In addition, hepcidin-mediated degradation of FPN indicates that P19 cells may respond to pro-inflammatory signaling and convert to select features of M1 macrophages. In summary, there are several research opportunities that may exploit the easily cultured P19 cell line, including study of macrophage-related iron handling behavior, possible identification of tumors using hepcidin as a biomarker, or modulation of the cancer phenotype by downregulating FPN^10^.

### MRI analysis

MRI is a promising tool for molecular imaging. Paramagnetic compounds, such as iron-based contrast agents, shorten longitudinal and transverse relaxation times (and hence increase relaxation rates) in the tissues where they accumulate. This results in a brightening of *T*_*1*_-weighted images and a darkening of *T*_*2*_-weighted images^49^. As such, the way cells handle iron in any tissue is expected to affect the MR signal. Having established that P19 cells may be a suitable model for the study of macrophage-like iron homeostasis^15^, we investigated the effect of iron supplementation (representing hemorrhage) and hepcidin (representing pro-inflammatory signaling) on MR transverse relaxation rates^15^.

All transverse relaxation rates increased after P19 cell culture in iron-supplemented medium and decreased within 4 hours of iron withdrawal. As expected, MRI faithfully tracks changes in total cellular iron content^14^ and, in particular, when regulation of iron export is involved. To mimic hepcidin-dependent alteration in iron homeostasis, the influence of hepcidin on MR transverse relaxation rates was also investigated. However, no significant changes in any of the transverse relaxation rates were observed. While this finding was consistent with the total cellular iron content measured in P19 samples, we considered whether downregulation of FPN might alter other aspects of intracellular iron handling not reflected by the magnitude of the MR signal.

The correlation between total cellular iron content and each transverse relaxation rate was examined for hepcidin-treated and control groups. This analysis exposed surprising differences in *R*_*2*_ and *R*_*2*_′ components of transverse relaxivity. In the absence of hepcidin, *R*_*2*_ displays a moderate correlation to total cellular iron (r = 0.473) while ${R}_{2}^{\text{prime}}$ displays a strong correlation (r = 0.719). However, the relationship was reversed in hepcidin-treated samples, with a strong correlation between total cellular iron and *R*_*2*_ (r = 0.866) and a weaker correlation to ${R}_{2}^{\text{prime}}$ (r = 0.532). These findings suggest that hepcidin treatment decreases the impact of ferritin on the static dephasing component of signal decay from microscopic inhomogeneities, typically observed as a decrease in ${R}_{2}^{\text{prime}}$. Thus, when FPN levels drop the irreversible *R*_*2*_ component increases. As a result of this change in iron metabolism arising from hepcidin-FPN interactions, the slopes of the best fit lines correlating transverse relaxation rate and cellular iron content were greater in hepcidin-treated groups compared to controls.

In this study, spin echo and gradient echo signals were acquired with relatively long first echo and inter-echo delays (for spin echo and gradient echo, the first TE = 13 ms and 6.12 ms, respectively). Therefore, if signal decay components with very rapid decay rates (T2 less than 1 ms) exist for these P19 cell samples, they would not have been detected by our acquisition. Very fast signal decay components that appear when cellular or tissue iron concentration becomes higher than we observed, for example as seen in liver^50^, could be detected using ultrashort TE^51–55^. However in our study the experimental signal decay points obtained fit well to a single exponential curve (Fig. 4b for gradient echo; data not shown revealed similar mono-exponential fits for the spin echo), demonstrating that these relaxation rate values should be good representations of, at the very least, signal decay components with time scales on the order of, or longer than, the first echo times.

Since total P19 cellular iron content did not change upon hepcidin treatment, other factor(s) may underlie these changes in the correlation between iron and relaxation rate. One hypothesis is that FPN degradation by hepcidin results in a re-arrangement of total cellular iron. For example, cellular iron is mainly available in two forms: an iron oxide present in ferritin, consisting of ferric ion (Fe III), and unbound iron present in the labile iron pool, mainly in the ferrous form (Fe II). The chemical state of iron (ferrous vs ferric) and its compartmentalization (free vs protein-bound) are two main factors that change when intracellular iron is redistributed. As a result, spin-spin interactions between iron particles and adjacent atoms may be affected and alter the contribution of reversible and irreversible components to the total transverse relaxation rate. For instance, Dietrich *et al*. examined solutions of ferric or ferrous chloride in phantoms at 3T^56^. They showed a significantly steeper slope of the line relating concentration of ferric ion and change in R2* compared to ferrous ion and R2*. These results support our findings and suggest that ionic form of iron in the labile iron pool influences transverse relaxivity. In the future, it may be useful to examine the relationship between transverse relaxation rates and measures of the labile iron pool^57^ or ferritin separately to better understand the impact of inflammatory signals like hepcidin on MRI.

Iron particles shorten *T*_*1*_ and *T*_*2*_, resulting in a signal increase in *T*_*1*_-weighted images (positive contrast) while producing a signal loss in *T*_*2*_-weighted images (negative contrast). While Liu *et al*.^15^ showed no influence of changes in extracellular iron on *T*_*1*_-weighted images in P19 cell phantoms, if hepcidin activity alters the LIP as proposed above, that might also influence *T*_*1*_ since it depends on the energy transfer between spins and the surrounding lattice. Thus, if chemical form of surrounding atoms or their compartmentalization is altered under different conditions of iron supplementation and hepcidin-mediated regulation of iron export, this might influence the efficiency of energy transfer and therefore, *T*_*1*_.

Our study in P19 cells uses an easily cultured cell line to examine iron export protein, the regulation of its activity by hepcidin, and the manner in which changes in total cellular iron influence MRI transverse relaxation rates. How well this multi-potent cell model reflects immune cell activity remains to be seen. The ability of P19 cells to differentiate down all 3 cell lineages does not necessarily encompass the spectrum of iron export activity seen in monocytes as they differentiate to macrophages. Possibly, the P19 cancer cell phenotype has some similarity to tumor-associated macrophages. However, in general, cultured cells do not reflect the complexity of *in vivo* signalling. Systemic hepcidin-ferroportin interactions may be tempered by local tissue activities. In this respect, *in vivo* detection of inflammatory cells will depend on the tissue’s baseline MR signal. Nonetheless, in acute myocardial infarction, inflammatory cells are dominant in cardiac tissue^32^. The cell phantom used in this study isolated MR signals from a single iron-exporting cell line, pointing to changes in the form of intracellular iron, rather than total iron content, in response to hepcidin. This regulatory mechanism (*i*.*e*. degradation of ferroportin) influenced the slope of the line correlating transverse relaxation rates and cellular iron. Application of this knowledge for the non-invasive detection of hepcidin-mediated, pro-inflammatory signalling requires further investigation.

## Conclusion

Iron handling mechanisms in different cell types may influence cell tracking with MRI. Using P19 cells to model the iron recycling activity of M2 anti-inflammatory macrophages, we examined the effect of different conditions of iron supplementation on level of FPN, total cellular iron content and MR signal. The effect of hepcidin on these measures also indicated the potential for modeling M1 pro-inflammatory macrophage iron handling behavior.

Addition and removal of an extracellular iron supplement confirmed the iron import and export abilities of P19 cells. While level of TfRc was relatively constant, the pattern of FPN was dynamic. Moreover, hepcidin treatment resulted in FPN degradation and a rise in ubiquitin, consistent with reported activity^16^. This resulted in a strong correlation between each transverse relaxation rate (*R*_*2*_, *R*_2_*) and total cellular iron content, with a higher slope in the line-of-best-fit compared to control cells. In the future, potential hepcidin-induced changes in the form of cellular iron may be used to non-invasively exploit (1) different macrophage phenotypes based on the control of iron export and (2) inflammation-related changes using MRI.

## Supplementary information

Supplementary Information.
