# Supplementary material for: Hepcidin-mediated Iron Regulation in P19 Cells is Detectable by Magnetic Resonance Imaging
Source: Sci Rep. 2020 Feb 21;10:3163. doi: 10.1038/s41598-020-59991-4 (PMC7035373; doi:10.1038/s41598-020-59991-4)
Supplement: Supplementary file 1 — Supplementary Information. [file 41598_2020_59991_MOESM1_ESM.pdf]

Hepcidin-mediated Iron Regulation in P19 Cells is Detectable by Magnetic Resonance Imaging

Kobra Alizadeh[1,2,3], *Qin Sun*[1,2,3], *Tabitha McGuire*[1], *Terry Thompson*[1,2,4,5], *Frank S. Prato*[1,2,3,4,5], *Jim Koropatnick*[6,7], *Neil Gelman*[1,2,4] *and Donna E. Goldhawk*[1,2,3,*]

1. Imaging, Lawson Health Research Institute, London, Ontario, Canada
2. Medical Biophysics, Western University, London, Ontario, Canada
3. Collaborative Graduate Program in Molecular Imaging, Western University, London, Ontario, Canada
4. Medical Imaging, Western University, London, Ontario, Canada
5. Physics and Astronomy, Western University, London, Ontario, Canada
6. London Regional Cancer Program, London, Ontario, Canada
7. Oncology, Western University, London, Ontario, Canada

*Corresponding Author: E-mail: dgoldhawk@lawsonimaging.ca.

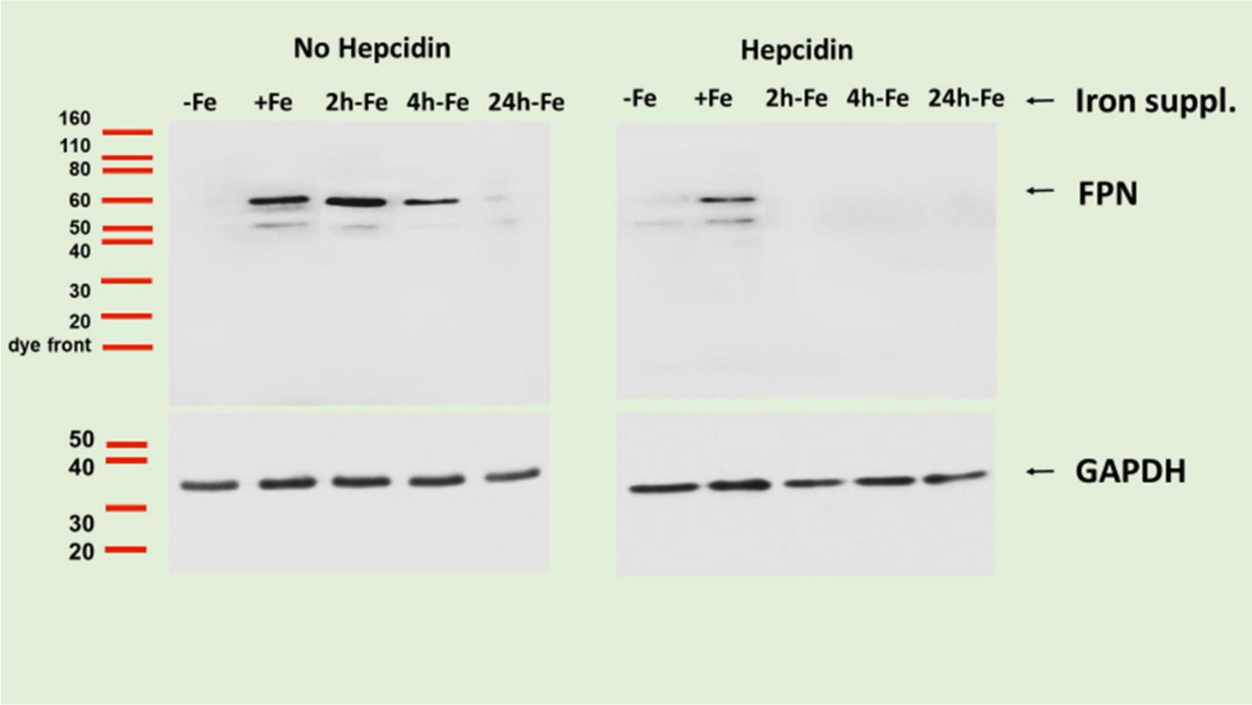

Figure S1. Level of ferroportin in P19 cells under various conditions of extracellular iron supplementation and hepcidin treatment. Cells were cultured either in non-supplemented (-Fe) or iron-supplemented medium (+Fe) for at least 5-7 days before iron supplementation withdrawal and an additional 1 (1h-Fe), 2 (2h-Fe), 4 (4h-Fe) or 24 (24h-Fe) hours of culture in non-supplemented medium, with or without 200 ng/ml hepcidin. Western blots of total cellular protein from these cell lysates were probed with rabbit α-FPN1. MW standards and dye front are shown in the left margin. Full length FPN protein is indicated with an arrow at approximately 63K. FPN degradation products are visible below the 63K band. Blots were stripped and reprobed with rabbit α-GAPDH to verify consistent protein loading in each lane. GAPDH is indicated with an arrow at approximately 37K.

a)

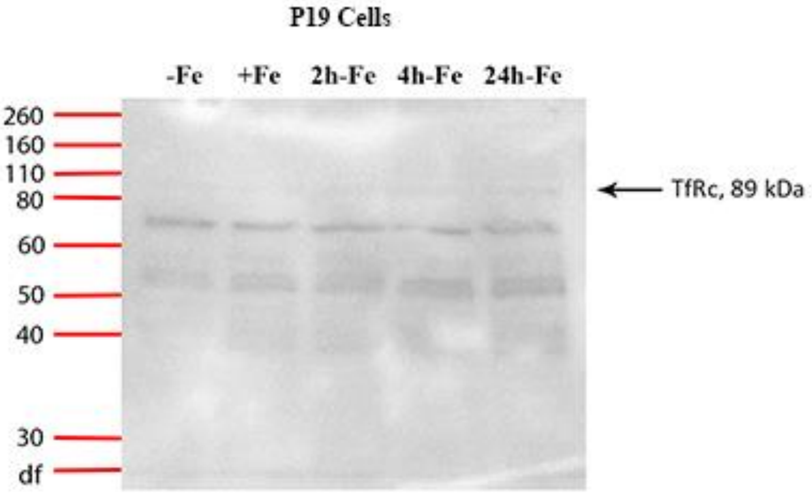
P19 Cells
-Fe
+Fe
2h-Fe
4h-Fe
24h-Fe
260
160
110
80
60
50
40
30
df
TfRc, 89 kDa

b)

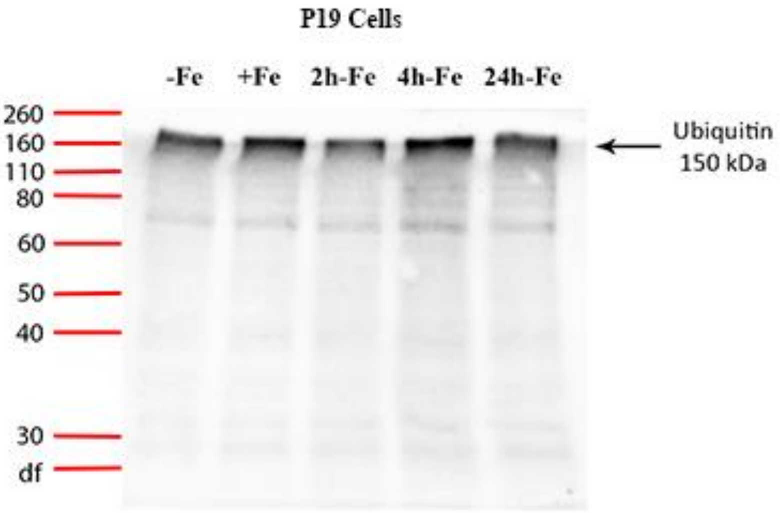
P19 Cells
-Fe
+Fe
2h-Fe
4h-Fe
24h-Fe
260
160
110
80
60
50
40
30
df
Ubiquitin
150 kDa

c)

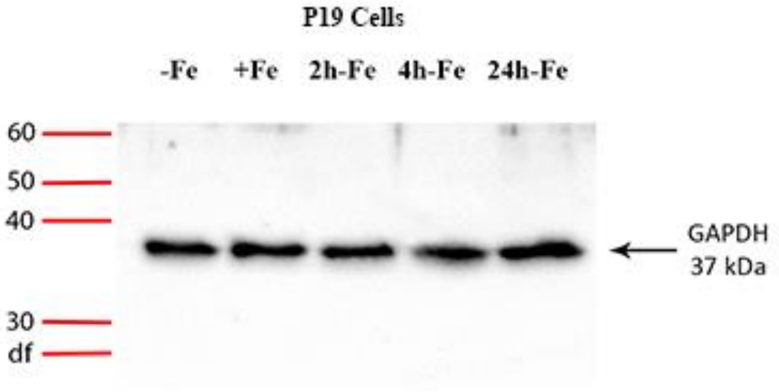
P19 Cells
-Fe
+Fe
2h-Fe
4h-Fe
24h-Fe
60
50
40
30
df
GAPDH
37 kDa

Figure S2. Level of transferrin receptor and ubiquitin in P19 cells under various conditions of extracellular iron supplementation. Cells were cultured either in non-supplemented (-Fe) or iron-supplemented medium (+Fe) for at least 5-7 days before iron supplementation withdrawal and an additional 1 (1h-Fe), 2 (2h-Fe), 4 (4h-Fe) or 24 (24h-Fe) hours of culture in non-supplemented medium. **(a)** A western blot of total cellular protein from these cell lysates was probed with rabbit α-TfRc. MW standards are shown in the left margin; df indicates the dye front. Full length TfRc protein is indicated with an arrow at approximately 89K. TfRc degradation products are visible below the 89K band. **(b)** The blot was stripped and reprobed with mouse α-Ubiquitin. Full length Ubiquitin protein is indicated with an arrow at approximately 150K. **(c)** Finally, the blot was stripped and reprobed with rabbit α-GAPDH, as indicated by the arrow at approximately 37K.

a)

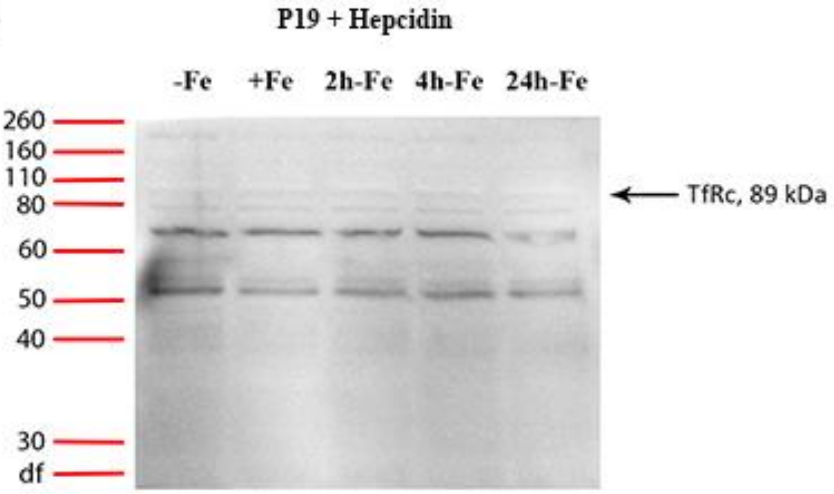
P19 + Hepcidin
-Fe
+Fe
2h-Fe
4h-Fe
24h-Fe
260
160
110
80
60
50
40
30
df
TfRc, 89 kDa

b)

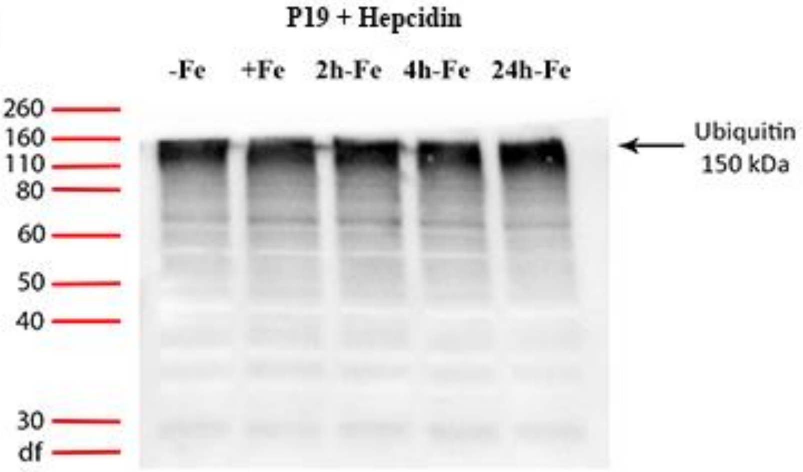
P19 + Hepcidin
-Fe
+Fe
2h-Fe
4h-Fe
24h-Fe
260
160
110
80
60
50
40
30
df
Ubiquitin
150 kDa

c)

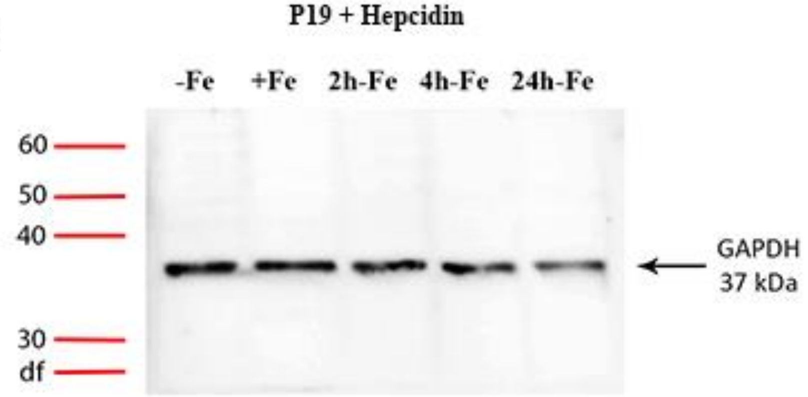
P19 + Hepcidin
-Fe
+Fe
2h-Fe
4h-Fe
24h-Fe
60
50
40
30
df
GAPDH
37 kDa

Figure S3. Level of transferrin receptor and ubiquitin in P19 cells responding to hepcidin treatment. Cells were cultured either in non-supplemented (-Fe) or iron-supplemented medium (+Fe) for at least 5-7 days before iron supplementation withdrawal and an additional 1 (1h-Fe), 2 (2h-Fe), 4 (4h-Fe) or 24 (24h-Fe) hours of culture in non-supplemented medium in the presence of 200 ng/ml hepcidin. In the case of hepcidin treatment +/-Fe, cells were incubated with hepcidin for the last 24 hours of culture. **(a)** A western blot of total cellular protein from these cell lysates was probed with rabbit α-TfRc. MW standards are shown in the left margin; df indicates the dye front. Full length TfRc protein is indicated with an arrow at approximately 89K. TfRc degradation products are visible below the 89K band. **(b)** The blot was stripped and reprobed with mouse α-Ubiquitin. Full length Ubiquitin protein is indicated with an arrow at approximately 150K. **(c)** Finally, the blot was stripped and reprobed with rabbit α-GAPDH, as indicated by the arrow at approximately 37K.

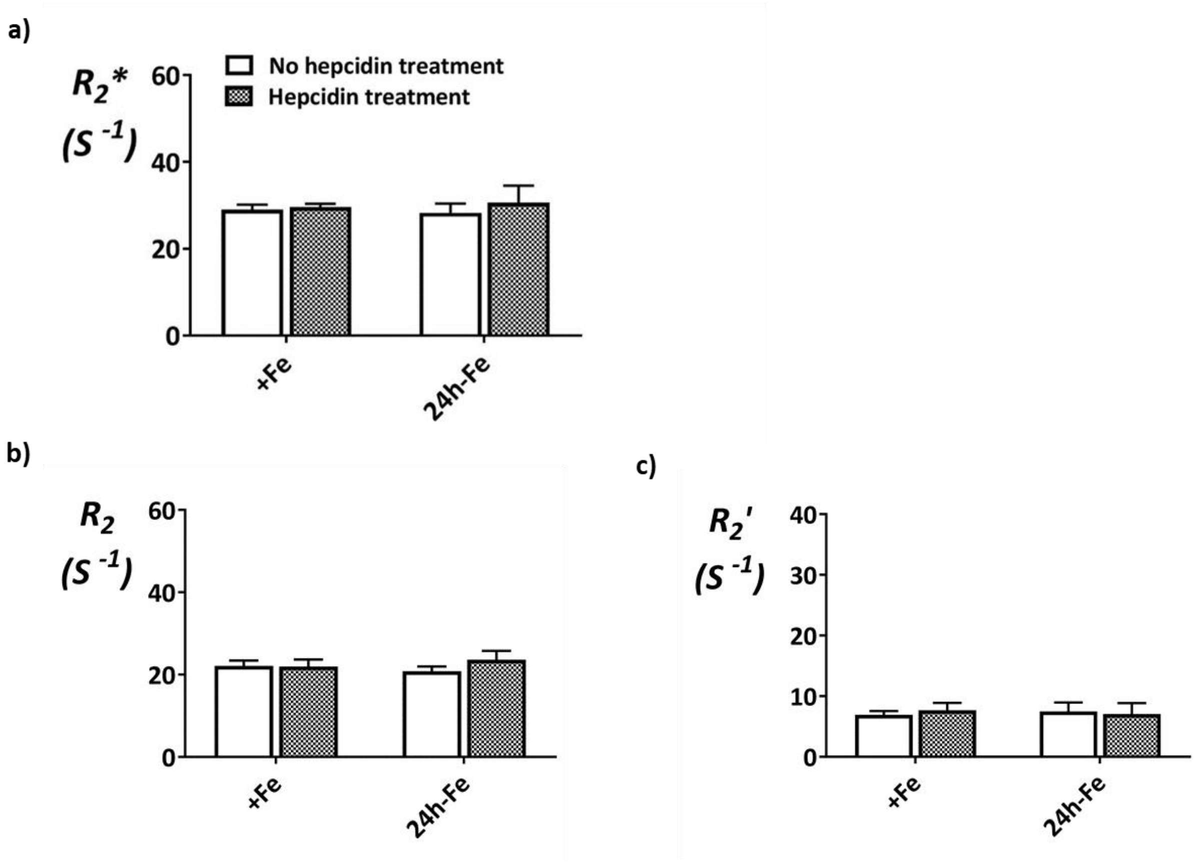

Figure S4. Transverse relaxation rates of P19 cells in response to hepcidin treatment. Cells were cultured in iron-supplemented medium (+Fe) for at least 5-7 days before withdrawal of iron supplementation and an additional 24 (24h-Fe) hours of culture in non-supplemented medium. Control samples (no hepcidin treatment) were compared to those incubated in the presence of hepcidin for the last 24 hours of their culture. Transverse relaxation rates for a) R_2* and b) R_2 were determined at 3T while R_2' (c) was calculated from the difference: $R_2' = R_2^* - R_2$. No difference was observed between hepcidin and non-hepcidin treatment groups. Data are the mean ± SEM: +Fe (no hepcidin), n=9; +Fe (hepcidin treatment), n=3; 24h-Fe (no hepcidin), n=9; 24h-Fe (hepcidin treatment), n=8.

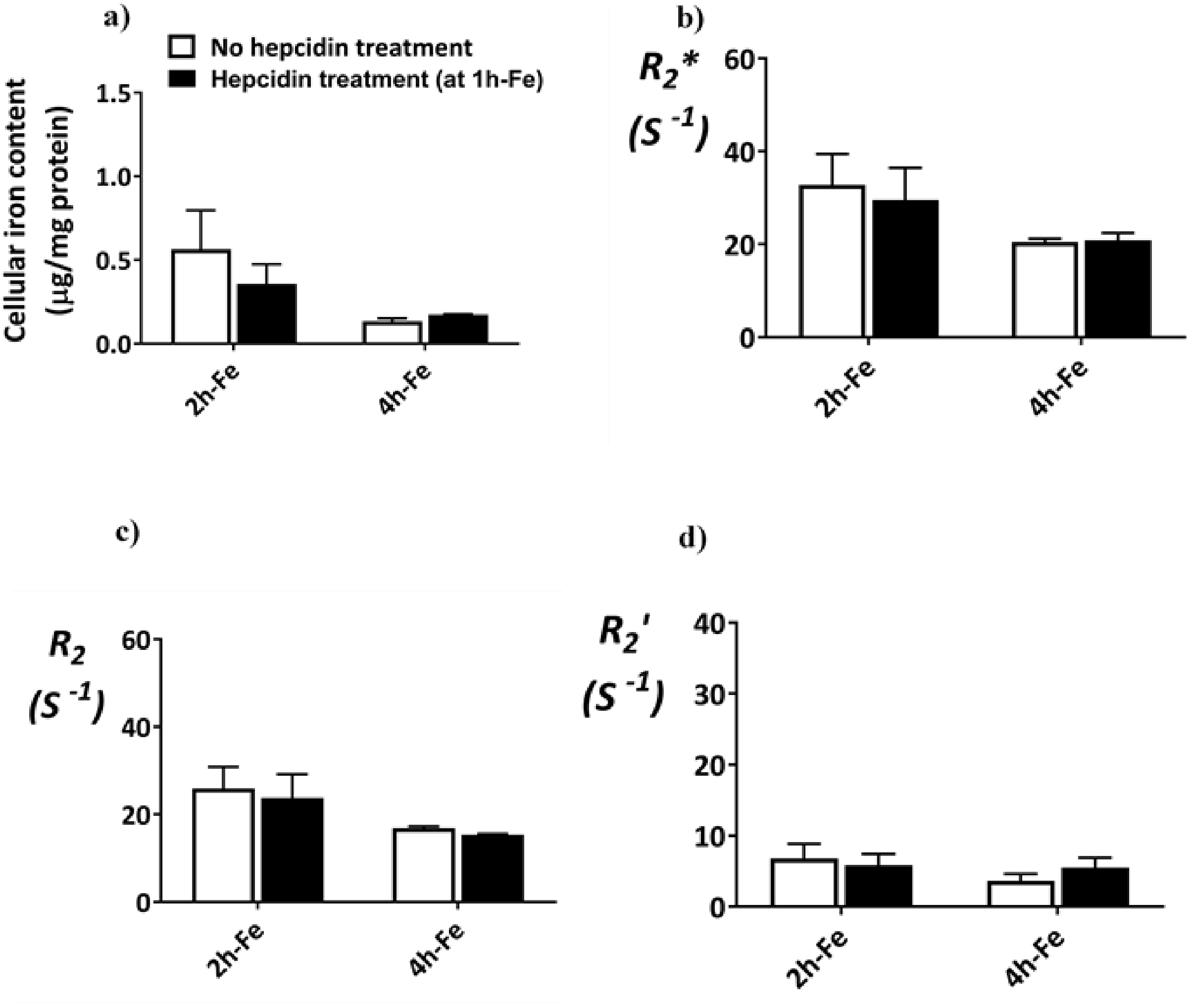

Figure S5. P19 response to hepcidin treatment. Cells were cultured in iron-supplemented medium (+Fe) for at least 5-7 days before supplementation withdrawal and an additional 2 (2h-Fe) or 4 (4h-Fe) hours of culture in non-supplemented medium with or without hepcidin addition at 1h-Fe. a) Total cellular iron content was measured by ICP-MS and normalized to total amount of protein (n=3). Transverse relaxation rates for b) R_2* and c) R_2 were determined at 3T while R_2' (d) was calculated from the difference: $R_2' = R_2* - R_2$. No difference was observed between hepcidin and non-hepcidin treatment groups. Data are the mean ± SEM for n=3.
